# Difference in risk of preterm and small-for-gestational-age birth depending on maternal occupations in Japan

**DOI:** 10.1186/s13104-023-06539-0

**Published:** 2023-10-05

**Authors:** Tasuku Okui

**Affiliations:** Medical Information Center, Kyushu University Hospital, Fukuoka city, Japan

**Keywords:** Small-for-gestational-age, Preterm birth, Japan, Maternal occupations

## Abstract

**Objectives:**

In this study, an association between the mother’s occupations with preterm and small-for-gestational-age (SGA) births was investigated using national data in Japan, and individual-level birth data from the Report of Vital Statistics: Occupational and Industrial Aspects in the 2015 fiscal year were used. Preterm and SGA birth rates were calculated for each of infant characteristics, and relative risk of each type of maternal occupations (categorized into 12 types) for the outcomes was estimated using a log binomial regression model.

**Results:**

Data of 997,600 singleton births were analyzed. Among maternal occupations, preterm birth rate was highest among carrying, cleaning, packaging, and related workers (5.65%) and lowest among security workers (4.24%). SGA birth rate was highest among manufacturing process workers (5.91%) and lowest among security workers (4.00%). We found significantly elevated risks for preterm birth among manufacturing process workers compared with unemployed mothers, and significantly elevated risks for SGA birth compared with unemployed mothers were observed among sales workers, service workers, and manufacturing process workers. In contrast, security workers had a significantly decreased risk for SGA birth compared with unemployed mothers.

**Supplementary Information:**

The online version contains supplementary material available at 10.1186/s13104-023-06539-0.

## Introduction

Preterm birth and small-for-gestational-age (SGA) in infants is a major adverse birth outcome. SGA rate differs largely depending on regions and tends to be high in developing countries [1, 2]. Although preterm birth rate globally stands at approximately 10% [2], the rate in Japan is much lower [3]. However, preterm birth is a risk factor for perinatal and infant mortality [4, 5], and adversely effects neurodevelopment or the incidence of cardiovascular diseases [6]. Infants with SGA, which is the other adverse birth outcome and a focus of epidemiological studies [7, 8], have an elevated risk of delayed development or childhood mortality [9–11]. Therefore, prevention of these adverse birth outcomes is a basic medical and societal need.

It is known that some characteristics of mothers, such as maternal age, smoking habits, and maternal socioeconomic status, are risk factors of adverse birth outcomes [12–14]. In Japan, epidemiological studies have shown that the mother’s pre-pregnancy body mass index (BMI) and the maternal level of education were risk factors for preterm and SGA births [15–18]. In addition, occupation is another major socioeconomic characteristic of parents. The association between maternal occupation and outcomes, such as infant mortality or low birth weight, has been investigated in Japan [19, 20]. However, there has been no systematic study for the relationship between the mother’s occupations and preterm and SGA births, using nationwide government statistics data in Japan. One study on maternal occupations in Sweden showed that “mechanics and iron and metalware workers” had a higher risk of giving birth to infants with SGA [21], and difference was also investigated and observed also in Korea and the United States [22, 23]. By identifying occupations that confer high risk for these adverse birth outcomes to parents also in Japan, we might be able to provide targeted support to people with those occupations at their workplace or in their communities.

To address this goal, we used the Vital Statistics data in Japan to investigate the association between maternal occupations and preterm and SGA births.

## Main text

### Methods

#### Data source and data preprocessing

We obtained nationwide individual-level data of the Report of Vital Statistics: Occupational and Industrial Aspects in the 2015 fiscal year from the Japanese Ministry of Health, Labor, and Wealth. Use of the data for research was permitted by the Ministry based on Article 33 of the Statistics Act. The survey for the report is conducted every 5 years, and the data include infants which were born between April 1, 2015 and March 31, 2016. The Report corresponds to the Vital Statistics with occupational and industry information. Vital Statistics is a survey by the Japanese government, and information from the birth certificates of all over the country are gathered by the Ministry of Health, Labor, and Wealth. It is mandatory for a parent to submit birth certificates of their children to a municipality within 14 days after childbirth. The birth certificate is a self-administered questionnaire, while health-related information, such as birthweight and gestational age, are written by physicians or midwives at the time of childbirth. The same data have been analyzed in previous studies in Japan [19, 24], and the maternal occupations at the time of birth were surveyed. We used the birth data, which included maternal age, maternal nationality, wedlock status, sex, birth weight, number of fetuses, gestational age, parity, household occupation, and mother’s occupation for each infant. Categories for maternal occupation in the data were 12 types, which was based on the Standard Occupational Classification for Japan. Household occupation means type of main occupation for each household, which is classified as farmer, self-employed worker, full-time worker 1, full-time worker 2, others, and unemployed. These six types of household occupations are the choices listed in the birth certificate for decades, and the parents must choose one from them.

Preterm birth was defined as gestational age below 37 weeks. SGA is often defined as a birth weight below the 10th percentile of the sex- and gestational age-specific standard reference value [8, 25]. In Japan, previous studies of SGA births generally used a sex- parity- and gestational age-specific standard reference for birthweight based on a Japanese neonatal anthropometric chart [7, 15, 26, 27]. Following this precedent, we defined SGA infants as those with birth weight below the 10th percentile of the same standard reference. For this study, we excluded multiples, and restricted the analysis to singleton infants. In addition, infants who were born between 22 and 41 weeks were used for the analysis of SGA births because the standard reference for birthweight in Japan covers only gestational ages of those periods [26].

### Statistical analysis

We tallied the number of births, preterm births, and SGA births and preterm and SGA birth rates for each infant characteristic. We defined six disjoint groups for maternal age (≤ 19 years, 20–24 years, 25–29 years, 30–34 years, 35–39 years, and ≥ 40 years), three groups for gestational age (< 32 weeks, 32–36 weeks, and ≥ 37 weeks), and three groups for birth weight (< 1500 g, 1500–2499 g, ≥2500 g). Parity for infants was categorized into primiparous and multiparous. Maternal nationality was classified into Japanese and non-Japanese. Chi-square test was conducted between each of the birth characteristics and each of the outcomes. In addition, the relative risk of preterm and SGA birth for each type of occupation was calculated using a log binomial regression model with other characteristics as covariates. The log binomial regression model is a method used for calculating the relative risks for binary data [28, 29]. Unemployed persons were used as a reference in the regression analysis for maternal occupations because the number of births was the largest. We reported the relative risk, its 95% confidence interval, and the p-value.

Complete-case analysis was conducted in the chi-square test and the regression analysis for dealing with missing data. In addition, multiple imputation was used as a sensitivity analysis for dealing with missing data in the regression analysis [30]. All statistical analyses were performed by using the statistical software R (version 4.6.1) [31]. In addition, statistics shown in this study were analyzed by the author using the data provided from the Ministry of Health, Labor and Wealth, and those are different from statistics published by the Ministry.

## Results

**Fig. 1:**
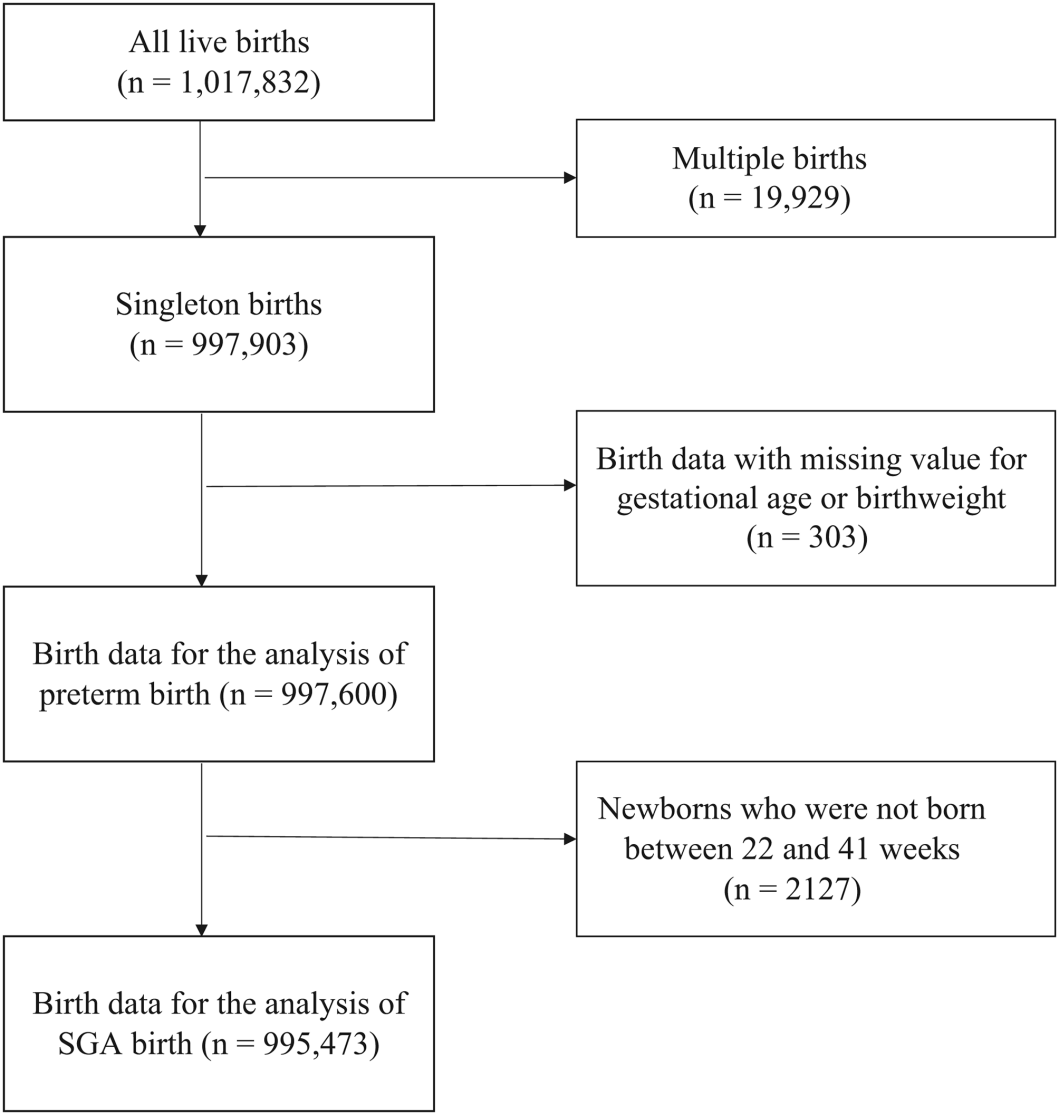
Flowchart of selecting birth data

Figure 1 shows the flowchart of selecting birth data for analysis.

Table 1 shows number of live births, preterm births, and SGA births and rates of preterm and SGA births for each of the characteristics. The greatest number of births was registered for unemployed persons among maternal occupations. The preterm and SGA rates were particularly high for mothers of age 40 and older. Among maternal occupations, preterm birth rate was highest among carrying, cleaning, packaging, and related workers (5.65%) and lowest among security workers (4.24%). SGA birth rate was highest among manufacturing process workers (5.91%) and lowest among security workers (4.00%). Most of the characteristics such as maternal occupations were statistically significantly associated with the birth outcomes.

**Table 1:** Number of live births, preterm births, and SGA births and rates of preterm and SGA births for each of the characteristics

		Preterm birth				SGA birth		
	Number of live births	Number	Rate (%)	p-value ^a^	Number of infants born between 22–41 weeks	Number	Rate (%)	p-value ^a^
Total	997,600	46,685	4.68		995,473	51,603	5.18	
Maternal age group				< 0.001				< 0.001
Under 20 years	11,907	659	5.53		11,882	624	5.25	
20–24 years	84,431	3,581	4.24		84,208	4,422	5.25	
25–29 years	259,444	10,230	3.94		258,890	13,185	5.09	
30–34 years	362,287	16,076	4.44		361,535	18,223	5.04	
35–39 years	225,745	12,401	5.49		225,282	11,939	5.30	
40 years or more	53,786	3,738	6.95		53,676	3,210	5.98	
Maternal nationality				< 0.001				< 0.001
Japanese	974,010	45,429	4.66		971,954	50,932	5.24	
Non-Japanese	23,590	1,256	5.32		23,519	671	2.85	
Sex				< 0.001				0.765
Female	486,483	19,836	4.08		485,398	25,123	5.18	
Male	511,117	26,849	5.25		510,075	26,480	5.19	
Parity				< 0.001				< 0.001
Primiparous	475,620	21,609	4.54		473,912	25,378	5.36	
Multiparous	521,980	25,076	4.80		521,561	26,225	5.03	
Wedlock status				< 0.001				< 0.001
In wedlock	972,910	44,949	4.62		970,864	49,944	5.14	
Out of wedlock	24,690	1,736	7.03		24,609	1,659	6.74	
Gestational age				< 0.001				< 0.001
< 32 weeks	5,672	5,672	100.00		5,666	1,173	20.70	
32–36 weeks	41,013	41,013	100.00		41,013	3,636	8.87	
>= 37 weeks	950,915	0	0.00		948,794	46,794	4.93	
Birth weight				< 0.001				< 0.001
1499 g or less	5,841	5,786	99.06		5,835	2,337	40.05	
1500–2499 g	75,561	23,763	31.45		75,546	29,988	39.70	
>=2500 g	916,198	17,136	1.87		914,092	19,278	2.11	
Household occupation				< 0.001				< 0.001
Farmer	12,789	608	4.75		12,755	642	5.03	
Self-employed worker	71,471	3,414	4.78		71,301	3,662	5.14	
Full-time worker 1 ^b^	332,563	15,703	4.72		331,852	17,859	5.38	
Full-time worker 2 ^c^	454,301	20,672	4.55		453,433	22,660	5.00	
Others	84,339	3,867	4.59		84,131	4,301	5.11	
Unemployed	20,160	1,277	6.33		20,106	1,309	6.51	
Missing	21,977	1,144	5.21		21,895	1,170	5.34	
Maternal occupation				0.001				< 0.001
Unemployed persons	535,812	25,135	4.69		534,739	27,325	5.11	
Administrative and managerial workers	5,140	254	4.94		5,129	245	4.78	
Professional and engineering workers	149,002	6,800	4.56		148,662	7,613	5.12	
Clerical workers	127,405	5,915	4.64		127,135	6,727	5.29	
Sales workers	32,548	1,495	4.59		32,467	1,776	5.47	
Service workers	64,555	3,092	4.79		64,385	3,475	5.40	
Security workers	2,904	123	4.24		2,901	116	4.00	
Agriculture, forestry, and fishery workers	4,132	183	4.43		4,124	197	4.78	
Manufacturing process workers	16,569	883	5.33		16,543	977	5.91	
Transport and machine operating workers	1,549	74	4.78		1,547	67	4.33	
Construction and mining workers	1,893	85	4.49		1,888	91	4.82	
Carrying, cleaning, packaging, and related workers	1,718	97	5.65		1,712	78	4.56	
Workers engaged in an unclassified occupation	12,833	555	4.32		12,800	656	5.13	
Missing	41,540	1,994	4.80		41,441	2,260	5.45	

SGA, small-for-gestational-age. a. p-value indicates the result of chi-square test for an association between an outcome and each variable. b. Full-time worker 1 means household of a full-time worker of a company or private shop (except for public offices) who has 1–99 employees. c. Full-time worker 2 means household of a board member or of a full-time worker who does not correspond with the full-time worker 1.

Table 2 shows analysis results of regression analysis showing relative risk of maternal occupations for preterm and SGA birth. We found significantly elevated risks for preterm birth among manufacturing process workers compared with unemployed mothers, and significantly elevated risks for SGA birth compared with unemployed mothers were observed among sales workers, service workers, and manufacturing process workers. In contrast, security workers had a significantly decreased risk for SGA birth compared with unemployed mothers. In addition, the relative risk was the highest level among manufacturing process workers both for preterm and SGA births.

**Table 2:** Analysis results of regression analysis showing relative risk of maternal occupations for preterm and SGA birth

	Preterm birth		SGA birth	
	RR (95% CI)	p-value	RR (95% CI)	p-value
Maternal age group				
Under 20 years	1.084 (0.999, 1.177)	0.053	0.932 (0.858, 1.013)	0.098
20–24 years	0.911 (0.877, 0.945)	< 0.001	0.991 (0.958, 1.026)	0.620
25–29 years	0.880 (0.858, 0.903)	< 0.001	0.992 (0.970, 1.015)	0.491
30–34 years	Reference		Reference	
35–39 years	1.232 (1.204, 1.262)	< 0.001	1.052 (1.028, 1.077)	< 0.001
40 years or more	1.558 (1.504, 1.614)	< 0.001	1.171 (1.128, 1.216)	< 0.001
Maternal nationality				
Japanese	Reference		Reference	
Non-Japanese	1.096 (1.034, 1.161)	0.002	0.532 (0.492, 0.575)	< 0.001
Sex				
Female	Reference		Reference	
Male	1.288 (1.264, 1.312)	< 0.001	1.003 (0.986, 1.020)	0.739
Parity				
Primiparous	Reference		Reference	
Multiparous	1.004 (0.986, 1.024)	0.645	0.930 (0.914, 0.947)	< 0.001
Wedlock status				
In wedlock	Reference		Reference	
Out of wedlock	1.398 (1.318, 1.482)	< 0.001	1.240 (1.169, 1.315)	< 0.001
Household occupation	Reference		Reference	
Farmer	1.062 (0.974, 1.158)	0.173	1.017 (0.935, 1.106)	0.694
Self-employed worker	1.013 (0.977, 1.050)	0.496	1.029 (0.994, 1.065)	0.110
Full-time worker 1 ^a^	1.046 (1.024, 1.067)	< 0.001	1.083 (1.062, 1.105)	< 0.001
Full-time worker 2 ^b^	Reference		Reference	
Others	1.024 (0.989, 1.059)	0.183	1.032 (0.999, 1.066)	0.058
Unemployed	1.182 (1.107, 1.262)	< 0.001	1.246 (1.169, 1.329)	< 0.001
Maternal occupation				
Unemployed persons	Reference		Reference	
Administrative and managerial workers	0.975 (0.863, 1.100)	0.678	0.909 (0.802, 1.030)	0.135
Professional and engineering workers	0.979 (0.953, 1.005)	0.112	1.003 (0.978, 1.029)	0.806
Clerical workers	0.975 (0.948, 1.003)	0.076	1.026 (0.999, 1.053)	0.060
Sales workers	0.985 (0.935, 1.037)	0.564	1.062 (1.013, 1.113)	0.013
Service workers	1.031 (0.993, 1.070)	0.106	1.043 (1.007, 1.080)	0.017
Security workers	0.951 (0.798, 1.134)	0.578	0.817 (0.684, 0.977)	0.027
Agriculture, forestry, and fishery workers	0.913 (0.782, 1.066)	0.249	0.956 (0.824, 1.110)	0.557
Manufacturing process workers	1.156 (1.082, 1.234)	< 0.001	1.178 (1.107, 1.254)	< 0.001
Transport and machine operating workers	1.029 (0.823, 1.288)	0.801	0.847 (0.669, 1.072)	0.167
Construction and mining workers	0.941 (0.763, 1.160)	0.570	0.952 (0.779, 1.164)	0.631
Carrying, cleaning, packaging, and related workers	1.173 (0.965, 1.426)	0.108	0.878 (0.705, 1.094)	0.247
Workers engaged in an unclassified occupation	0.901 (0.828, 0.981)	0.017	0.987 (0.912, 1.067)	0.736

SGA, small-for-gestational-age; RR, relative risk; CI, confidence interval. a. Full-time worker 1 means household of a full-time worker of a company or private shop (except for public offices) who has 1–99 employees. b. Full-time worker 2 means household of a board member or of a full-time worker who does not correspond with the full-time worker 1.

Supplementary Table 1 shows analysis results of regression analysis showing relative risk of maternal occupations for preterm and SGA birth using multiple imputation. Similar results were obtained as the main analysis, while a significant risk ratio for SGA birth was not observed for sales and service workers.

## Discussion

An association between the adverse birth outcomes and maternal occupations was revealed from an analysis of nationwide Vital Statistics data in Japan. Specifically, we have found that the risk of preterm and SGA births varied depending on maternal occupations. We now discuss the possible reasons for those associations.

Regarding the mother’s occupation, it was suggested that the risk for manufacturing process workers was the highest. The elevated risk for adverse birth outcomes among manufacturing process workers might indirectly reflect the prevalence of lower socioeconomic status among Japanese female manufacturing process workers. It is known that, in Japan, the proportion of university graduates is relatively low and the proportion of junior high school or elementary school graduates is relatively high for manufacturing process workers in the occupational classifications among women aged 20–49 years old [32]. The association of maternal education level or socioeconomic status with preterm birth and SGA is known [33, 34]. Specifically, lower socioeconomic status of the mother is positively associated with higher rate of preterm and SGA births in Japan [16, 18]. Furthermore, lower socioeconomic status is associated with the presence of medical risk factors, such as high smoking rate and low utilization of prenatal care, for adverse birth outcomes in Japan [35, 36]. However, the risk was not elevated among other types of workers whose educational level were relatively low, such as transport and machine operating workers, indicating that education level and socioeconomic status are not sufficient to fully explain the observations. Instead, other factors, such as employment status or occupation-specific factors could, in part, explain the elevated risk for preterm and SGA births among manufacturing process workers or other workers (service workers and sales workers). For example, long standing time at work or frequent night shifts are also known to be associated with the adverse birth outcomes [37–39], and it might have affected the results. In addition, security workers had the lowest risk for SGA births. As one possibility, physical characteristics of those workers might be related to the results. It is known that maternal height or BMI affects SGA births, and lower height or BMI is associated with higher risk of SGA birth [40–42]. Generally, adequate body weight and height are required for becoming security workers, such as police officers and self-defense officers, and it might have favorable effects on birth outcomes. An association of maternal occupations with socioeconomic and physical characteristics is needed to be surveyed in order to identify the reasons for the difference in risks.

One strength of this study is that we used a large set of nationwide birth data for the analysis, and the study results represent nationwide trends in Japan. In particular, the association between maternal occupations and SGA and preterm birth is demonstrated for the first time using nationwide Vital Statistics. We specifically highlighted a higher risk of preterm birth and SGA for mothers employed as manufacturing process workers. This finding has important implications. First, future studies are needed to investigate the causes for the observed association. Second, there is a need for implementing effective prevention measures to lower the risk of adverse birth outcomes. Adjusting the work load or improving working conditions might be needed to lower those risks for pregnant manufacturing process workers. A call for attention in working places or prenatal care for pregnant women with those occupations might help. Finally, providing health guidance targeted to husbands of pregnant women whose occupations have higher risk of adverse birth outcomes might be also effective.

### Limitations

One limitation of this study is the lack of data on women’s behaviors or physical characteristics, such as smoking status, BMI, utilization of prenatal care, chronic diseases, and working hours, that are known risk factors for adverse birth outcomes. Another limitation is the indirect inference of maternal educational level or physical characteristics because of the absence of such data in the dataset we analyzed. Socioeconomic status indicators of a mother, such as educational level or household income were not available in the data, and we could not fully differentiate the effect of maternal occupations from that of maternal socioeconomic status. Thus, to properly account for those factors that the survey does not collect, an epidemiological study designed to target those factors needs to be conducted in the future.

### Electronic supplementary material

Below is the link to the electronic supplementary material.

Supplementary Material 1

## Data Availability

The data that support the findings of this study are available from the Ministry of Health, Labour, and Welfare in Japan. However, restrictions apply to the availability of these data, which were provided under license for the current study.

## List of abbreviations

SGA: small-for-gestational-age
RR: relative risk
CI: confidence interval
BMI: body mass index
