# Supplementary material for: Difference in risk of preterm and small-for-gestational-age birth depending on maternal occupations in Japan
Source: BMC Res Notes. 2023 Oct 5;16:259. doi: 10.1186/s13104-023-06539-0 (PMC10557295; doi:10.1186/s13104-023-06539-0)
Supplement: Supplementary file 1 — Supplementary Material 1 [file 13104_2023_6539_MOESM1_ESM.docx]

Supplementary table 1. Analysis results of regression analysis showing relative risk of maternal occupations for preterm and SGA birth using multiple imputation

|  | Preterm birth | | SGA birth | |
| --- | --- | --- | --- | --- |
|  | RR (95% CI) | p-value | RR (95% CI) | p-value |
| Maternal age group |  |  |  |  |
| Under 20 years | 1.072 (0.990, 1.160) | 0.086 | 0.906 (0.835, 0.982) | 0.016 |
| 20-24 years | 0.914 (0.881, 0.949) | <0.001 | 0.992 (0.959, 1.025) | 0.617 |
| 25-29 years | 0.883 (0.862, 0.905) | <0.001 | 0.995 (0.974, 1.018) | 0.681 |
| 30-34 years | Reference |  | Reference |  |
| 35-39 years | 1.236 (1.208, 1.264) | <0.001 | 1.054 (1.030, 1.078) | <0.001 |
| 40 years or more | 1.555 (1.502, 1.610) | <0.001 | 1.181 (1.138, 1.225) | <0.001 |
| Maternal nationality |  |  |  |  |
| Japanese | Reference |  | Reference |  |
| Non-Japanese | 1.101 (1.042, 1.163) | <0.001 | 0.526 (0.487, 0.567) | <0.001 |
| Sex |  |  |  |  |
| Female | Reference |  | Reference |  |
| Male | 1.289 (1.266, 1.312) | <0.001 | 1.003 (0.986, 1.020) | 0.713 |
| Parity |  |  |  |  |
| Primiparous | Reference |  | Reference |  |
| Multiparous | 1.006 (0.987, 1.024) | 0.560 | 0.930 (0.914, 0.947) | <0.001 |
| Wedlock status |  |  |  |  |
| In wedlock | Reference |  | Reference |  |
| Out of wedlock | 1.420 (1.343, 1.502) | <0.001 | 1.256 (1.186, 1.330) | <0.001 |
| Household occupation | Reference |  | Reference |  |
| Farmer | 1.049 (0.891, 1.234) | 0.565 | 0.999 (0.875, 1.140) | 0.984 |
| Self-employed worker | 1.017 (0.975, 1.061) | 0.433 | 1.033 (0.992, 1.075) | 0.114 |
| Full-time worker 1 ^a^ | 1.041 (1.018, 1.065) | <0.001 | 1.082 (1.059, 1.105) | <0.001 |
| Full-time worker 2 ^b^ | Reference |  | Reference |  |
| Others | 1.026 (0.989, 1.064) | 0.171 | 1.036 (1.001, 1.072) | 0.046 |
| Unemployed | 1.160 (1.084, 1.241) | <0.001 | 1.240 (1.157, 1.330) | <0.001 |
| Maternal occupation |  |  |  |  |
| Unemployed persons | Reference |  | Reference |  |
| Administrative and managerial workers | 0.968 (0.824, 1.137) | 0.691 | 0.925 (0.781, 1.095) | 0.363 |
| Professional and engineering workers | 0.971 (0.937, 1.006) | 0.101 | 1.000 (0.964, 1.037) | 0.986 |
| Clerical workers | 0.972 (0.935, 1.011) | 0.155 | 1.028 (0.989, 1.068) | 0.162 |
| Sales workers | 0.987 (0.926, 1.052) | 0.689 | 1.060 (0.994, 1.131) | 0.074 |
| Service workers | 1.030 (0.981, 1.080) | 0.232 | 1.048 (0.999, 1.099) | 0.057 |
| Security workers | 0.943 (0.772, 1.154) | 0.570 | 0.779 (0.613, 0.988) | 0.040 |
| Agriculture, forestry, and fishery workers | 0.929 (0.764, 1.128) | 0.455 | 0.976 (0.818, 1.164) | 0.784 |
| Manufacturing process workers | 1.145 (1.044, 1.255) | 0.004 | 1.171 (1.069, 1.283) | <0.001 |
| Transport and machine operating workers | 1.031 (0.781, 1.361) | 0.827 | 0.850 (0.637, 1.135) | 0.270 |
| Construction and mining workers | 0.938 (0.721, 1.220) | 0.633 | 0.926 (0.730, 1.175) | 0.527 |
| Carrying, cleaning, packaging, and related workers | 1.226 (0.948, 1.587) | 0.120 | 0.921 (0.644, 1.319) | 0.652 |
| Workers engaged in an unclassified occupation | 0.911 (0.820, 1.013) | 0.084 | 1.019 (0.913, 1.137) | 0.738 |
| SGA, small-for-gestational-age; RR, relative risk; CI, confidence interval | |  |  |  |
| a. Full-time worker 1 means household of a full-time worker of a company or private shop (except for public offices) who has 1-99 employees | | | | |
| b. Full-time worker 2 means household of a board member or of a full-time worker who does not correspond with the full-time worker 1. | | | | |
